# Low temperature plasma enhanced CVD epitaxial growth of silicon on GaAs: a new paradigm for III-V/Si integration

**DOI:** 10.1038/srep25674

**Published:** 2016-05-11

**Authors:** Romain Cariou, Wanghua Chen, Jean-Luc Maurice, Jingwen Yu, Gilles Patriarche, Olivia Mauguin, Ludovic Largeau, Jean Decobert, Pere Roca i Cabarrocas

**Affiliations:** 1 LPICM-CNRS, Ecole Polytechnique, Université Paris-Saclay, 91128 Palaiseau, France; 2 III-V lab a joint laboratory between Alcatel-Lucent Bell Labs France, Thales Research and Technology and CEA-LETI, route de Nozay, 91460 Marcoussis, France; 3 LPN-CNRS, Route de Nozay, 91460 Marcoussis, France

## Abstract

The integration of III-V semiconductors with silicon is a key issue for photonics, microelectronics and photovoltaics. With the standard approach, namely the epitaxial growth of III-V on silicon, thick and complex buffer layers are required to limit the crystalline defects caused by the interface polarity issues, the thermal expansion, and lattice mismatches. To overcome these problems, we have developed a reverse and innovative approach to combine III-V and silicon: the straightforward epitaxial growth of silicon on GaAs at low temperature by plasma enhanced CVD (PECVD). Indeed we show that both GaAs surface cleaning by SiF_4_ plasma and subsequent epitaxial growth from SiH_4_/H_2_ precursors can be achieved at 175 °C. The GaAs native oxide etching is monitored with *in-situ* spectroscopic ellipsometry and Raman spectroscopy is used to assess the epitaxial silicon quality. We found that SiH_4_ dilution in hydrogen during deposition controls the layer structure: the epitaxial growth happens for deposition conditions at the transition between the microcrystalline and amorphous growth regimes. SIMS and STEM-HAADF bring evidences for the interface chemical sharpness. Together, TEM and XRD analysis demonstrate that PECVD enables the growth of high quality relaxed single crystal silicon on GaAs.

Silicon is the most widely used material in microelectronics, thanks to its numerous advantages: small mass density, good thermal conductivity, excellent passivation by SiO_2_, abundance and low cost, non-toxicity, maximum wafer diameter, tremendous amount of research, mature industrial processes, etc. On the other hand, III-V semiconductors feature unique and tunable optical and electronic properties, the ability to efficiently emit and detect light, etc. Likewise, Silicon and III-V materials have complementary properties that can advantageously be combined in photonics or photovoltaic devices^1^.

In this later field, crystalline silicon is the most widely used material and record power conversion efficiencies up to 25.6% have been reported^2^. A proven way to reach even higher efficiencies consists in stacking p-n diodes in series with different bandgaps spanning over a broad solar spectrum range. Usually made of III-V compounds, such multi-junction solar cells can reach significantly higher power conversion: the world record of 46.1% under concentration was recently achieved by the Fraunhofer ISE^3^. However the III-V compounds remain expensive and relatively scarce. Thus a hybrid PV device combining the advantages of III-V multi-junction solar cells with the benefits of Si as the most wide-spread photovoltaic material is a highly sought-after goal. Indeed the band gap of crystalline silicon is well adapted for multi-junctions: this material can potentially reach very high efficiencies when used in tandem, triple and quadruple junction solar cells in combination with III-V materials. For instance, a 1.12/1.74 eV tandem can theoretically reach 41.9% efficiency as calculated by Connolly *et al*.^4^, and in the case of a Ga_0.51_In_0.49_P/GaAs/Si triple junction, the efficiency goes up to 46.9% under 1 sun^5^.

The classic approach for combining III-V and silicon consists in the epitaxial growth of III-V layers on top of silicon wafers^6^,^7^,^8^. However, such hetero-epitaxial growth encounters two major issues: (i) the difference in lattice constant. The variety of gaps available in the III-V family covers a wide range of lattice parameters, but very few III-V compounds are lattice matched to silicon (e.g. GaAs and Si have about 4% lattice mismatch). (ii) The difference in thermal expansion coefficient. For more than 30 years, researchers have attempted to combine III-V and silicon, thus the issues are well documented^9^,^10^,^11^, and some of them are already well addressed. For example, the polar/non-polar interface of GaAs/Si system which creates defects such as anti-phase domains. However this problem was successfully solved by using Si substrates etched by 4–6° from the (100) plane^12^,^13^. But the two previously mentioned lattice and thermal mismatches are more serious issues. Indeed, the lattice mismatch results in a high density of dislocations and other defects in the growing crystal^14^. Moreover, as GaAs growth is usually performed at high temperature (e.g. 600–700 °C in MOCVD reactors), additional defects appear upon cooling the sample down to room temperature, due to the thermal expansion mismatch with silicon; those defects have deleterious effects on the device properties^15^,^16^. Another approach being explored to limit the impact of crystal defects is to grow buffer layers between the Si substrate and the active III-V material; it helps to reduce and relax the defects arising from the change of lattice constant. GaAsP or SiGe materials are under focus for such buffer layers^17^,^18^,^19^, however the density of threading dislocations typically achieved (~10^7^ cm^−2^) is deleterious for minority carrier devices, thus this approach remains challenging. Alternatively non-epitaxial techniques such as wafer bonding can be used to join semiconductor crystals with arbitrary lattice parameters^1^,^20^, but it also comes with great challenges regarding the surface quality.

In this study, we propose a completely new and simpler approach to combine Si and III-V: using plasma enhanced CVD epitaxy^21^,^22^,^23^ we have successfully grown silicon monocrystalline layers on GaAs at temperatures below 200 °C. By doing so, thermal expansion and diffusion issues are strongly reduced, and the polarity problems are completely suppressed as well, since non-polar Si is grown on polar GaAs. Epitaxial growth conditions and layer crystal quality are studied in details here. Although this approach relies on relatively expensive III-V substrates (e.g. GaAs), as opposed to III-V growth on silicon, the use of sacrificial layers and substrate reuse can drastically lower this additional cost^24^,^25^. In the field of Photovoltaics, this new approach can be advantageously used to grow inverted III-V(MOCVD)/SiGe(PECVD) multijunctions solar cells^26^,^27^.

## Results

### GaAs native oxide etching

**Figure 1:**
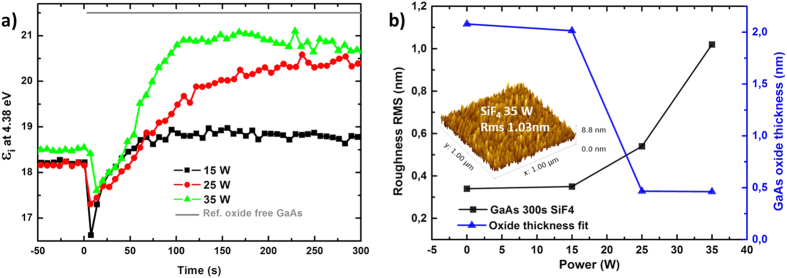
(**a**) *In-situ* real time spectroscopic ellipsometry monitoring of (001)-oriented GaAs native oxide removal by SiF_4_ plasma. t = 0 s corresponds to plasma ignition, and *ε*_*i*_ at 4.38 eV is monitored for three RF powers: 15 W (squares), 25 W (circles) and 35 W (triangles). (**b**) AFM RMS roughness (squares), measured *ex-situ* after 200 s SiF_4_ plasma treatment with different powers. The 35 W treatment results in a 1 nm roughness, as shown in the inset. Right axis: GaAs surface oxide (triangles) thickness deduced from fitting *ex-situ* ellipsometry measurements.

We have investigated experimentally the GaAs native oxide etching with SiF_4_ gas precursor using a PECVD reactor. The evolution of GaAs (001)-oriented wafer surface was monitored *in-situ* during the plasma etching by means of real time spectroscopic ellipsometry. The amplitude of the imaginary part of the pseudo-dielectric function (*ε*_*i*_) at 4.4 eV as a function of etching time for three values of plasma RF-power is shown in (Fig. 1a). The GaAs absorption depth at 4.4 eV being only 8 nm, the variations of *ε*_*i*_ at this energy are related to surface modifications. As a reference, the theoretical *ε*_*i*_ value for GaAs oxide free surface, at the substrate temperature of 180 °C, is shown by a grey line at the top of (Fig. 1a). Three RF-power conditions are tested for GaAs oxide etching: 15 W (squares), 25 W (circles) and 35 W (triangles). The etching plasma was switched on at t = 0 s, thus *ε*_*i*_(t < 0 s) corresponds to the GaAs surface with its native oxide. The net increase of *ε*_*i*_ with etching time, beyond the initial drop, indicates that the surface oxide is being removed. The best recipe turns out to be at 35 W, since this curve gets significantly closer to the expected theoretical *ε*_*i*_ as compared to lower RF-power recipes. For the 35 W curve, a maximum *ε*_*i*_ is reached after about 180–200 s of surface cleaning and then further plasma exposure results in surface degradation, most likely due to roughness increase. The root mean square roughness (RMS) measured *ex-situ* by atomic force microscope (AFM) after a 200 s SiF_4_ plasma for difference RF-power, is shown in (Fig. 1b) (square symbols). From an initial value below 0.4 nm for the out-of-the-box wafer surface, the 35 W treatment creates 1 nm roughness (see surface morphology in inset), which remains reasonable for subsequent epitaxial growth. The GaAs oxide thickness, as deduced from post-deposition ellipsometry measurements, is reduced below 0.5 nm by the 35 W treatment, as compared to the initial value slightly above 2 nm. The non-zero value for the oxide thickness after plasma cleaning is probably linked to oxide re-growth since the sample is measured in air. Overall, this is the proof that GaAs native oxide can be efficiently removed by *in-situ* SiF_4_ plasma at 175 °C. The mechanism involved is probably due to a mix of chemical reactions and sputtering effect.

### PECVD epitaxial growth

**Figure 2:**
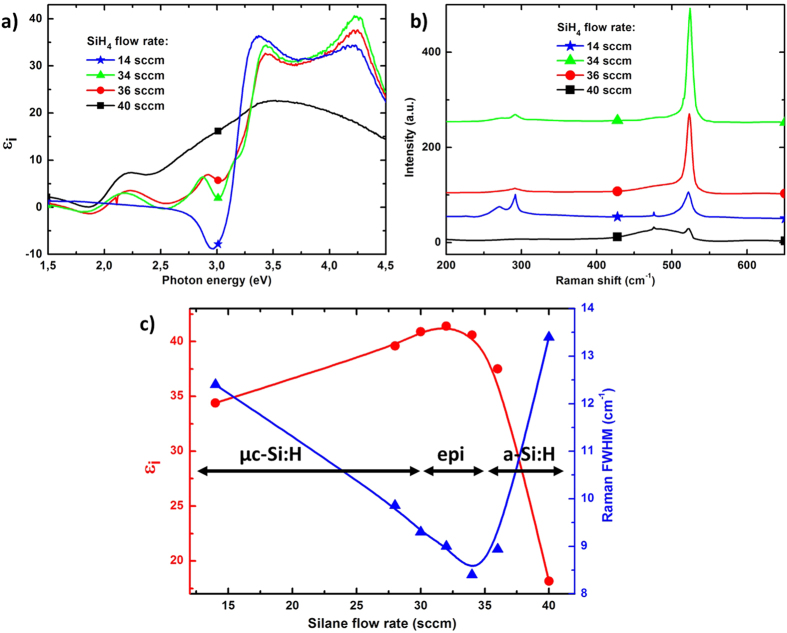
(**a**) Pseudo-dielectric function *ε*_*i*_ of PECVD silicon layers deposited from SiH_4_/H_2_ on plasma cleaned GaAs wafers with various silane flow rate: 14 sccm (stars), 34 sccm (triangles), 36 sccm (circles) and 40 sccm (squares). (**b**) Corresponding Raman spectra. (**c**) Left *ε*_*i*_ at 4.2 eV (circles) as a function of the silane flow rate. Right: FWHM of the Raman c-Si peak FWHM as a function of silane flow rate (triangles).

Once the optimum surface cleaning conditions were established, we have optimized the silane dilution in H_2_, since for PECVD silicon deposition, it influences strongly the crystalline structure of the layer. We have thus deposited a series of Si films on GaAs substrates, after the optimized 35 W SiF_4_ plasma cleaning step had been applied, in which the silane flow rate was varied while keeping all other parameters constant. The samples were analyzed after deposition by ellipsometry and Raman spectroscopy, as shown in (Fig. 2a,b). The *ε*_*i*_ is displayed in (Fig. 2a) for four silane flow rates: 14 sccm (stars), 34 sccm (triangles), 36 sccm (circles) and 40 sccm (squares). The same crystalline quality transition than in PECVD Si homoepitaxial growth is observed^28^: i) at high silane flow rate (40 sccm), the material is amorphous, ii) at moderate flow rate (34–36 sccm), the material is monocrystalline (as judged by *ε*_*i*_ characteristic peaks at 3.4 and 4.2 eV) and iii) at low silane flow rate (high dilution in H_2_), the Si layer on GaAs is microcrystalline (lower *ε*_*i*_ 4.2 eV peak compared to 34–36 sccm curves). In addition, the 14 sccm sample corresponds here to only 18 nm of Si on GaAs, while the 36 sccm sample, for the same deposition time, consists of 126 nm of Si on GaAs. One would expect lower *ε*_*i*_ peak amplitude for the 14 sccm samples with longer deposition time, since the substrate contribution to the signal would be smaller when buried under a thicker layer.

These structural transitions with silane dilution are further confirmed by Raman spectroscopy analysis. The spectra measured on the same series of samples are shown in (Fig. 2b). For the 40 sccm sample, the c-Si peak around 520 cm^−1^ is very small and dominated by a large shoulder centered at 480 cm^−1^, betraying the large a-Si:H fraction in this material; for the 14 sccm sample, no broad a-Si:H shoulder is detected, but the peak FWHM is quite large (12.5 cm^−1^). Note that both TO and LO GaAs modes are well detected at 268 and 292 cm^−1^, since the 14 sccm sample is very thin. The 34 and 36 sccm samples have a well-defined sharp c-Si peak, with 34 sccm exhibiting the smallest FWHM (8.4 cm^−1^). These results are summarized in (Fig. 2c) where the *ε*_*i*_ peak amplitude at 4.2 eV (circles) is plotted as a function of silane flow rate, and the right y-axis shows the Raman c-Si peak FWHM for the different silane flow rates (triangles). The maximum of *ε*_*i*_, around 41 at 4.2 eV, happens for nearly the same silane flow rate, namely 32 sccm, than the minimum Raman FWHM. The small discrepancy observed in optimum silane flux by ellipsometry and Raman (32 sccm and 34 sccm respectively) can be explained by the sensitivity of *ε*_*i*_ at 4.2 eV to the surface quality (contamination, oxide, roughness, etc.). Thus these two independent measurement techniques confirm the existence of an optimum silane dilution for low temperature heteroepitaxial growth of Si on GaAs: the monocrystalline growth regime happens for deposition conditions at the transition between microcrystalline and amorphous growth. We found the optimum crystal quality for a SiH_4_/(SiH_4_ + H_2_) of ~6%. Interestingly enough, this optimum is exactly the same as in the silicon homoepitaxial case^28^.

### Crystal quality assessment

**Figure 3:**
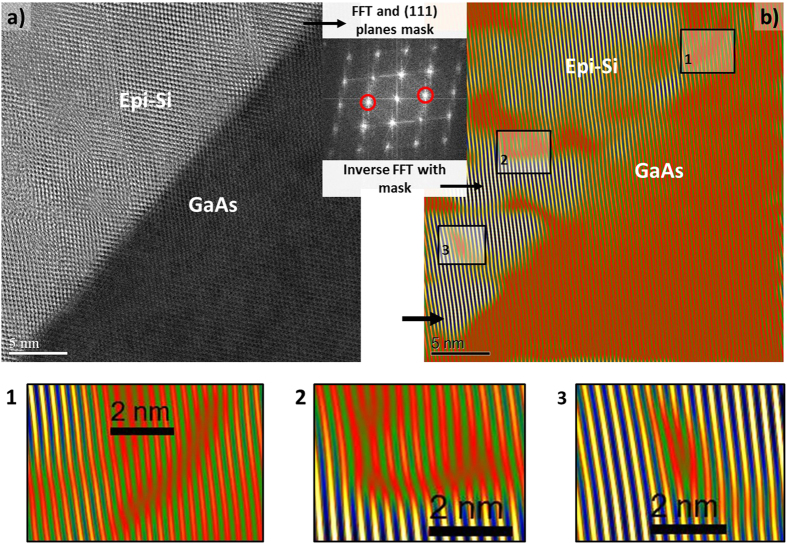
(**a**) High resolution STEM cross section along^1^,^2^,^3^,^4^,^5^,^6^,^7^,^8^,^9^,^10^ axis of PECVD epi-Si/GaAs interface. A mask is applied to the Fourier transformed image for selecting (111) planes, and the TEM image (**b**) is reconstructed by inverse FFT algorithm keeping only the contribution of (111) planes. 1, 2 and 3 are zoom on crystal defects.

The successful epitaxial growth at 175 °C of silicon on GaAs has been confirmed by both Raman and ellipsometry. To gain more insight into such heteroepitaxial layers crystal quality, we have performed cross section TEM/STEM analysis of the epi-Si/GaAs samples grown with the best silane dilution of 6%, as presented in the previous section. A STEM PECVD epi-Si/GaAs cross section along the [110] axis is detailed in (Fig. 3a). The interface appears sharp and well defined, and good atomic order can be distinguished in epi-Si layer. Despite the 4% lattice mismatch between the two materials, we could not detect threading dislocations with this technique. These first observations validate the benefit of a low temperature growth approach, which reduces thermal expansion related defects. Starting from a HR-STEM image of the interface, we could highlight the presence of defects lying in the (111) planes. First we have used a fast Fourier transform algorithm (FFT) to process the real-space image of the interface. The result, as shown in (Fig. 3a) inset, is conceptually equivalent to an electronic diffraction pattern, and thus reveals well defined spots corresponding to the contribution of crystallographic planes. Then by applying a mask on specific 111 spots in the FFT image and then performing inverse FFT algorithm, we could reconstruct a high resolution real-space image with color scale of the interface (Fig. 3b), but keeping only the contribution of the selected (111) planes. Like so, images (Fig. 3a,b) correspond to the exact same interface area. The insets 1, 2 and 3 correspond to zooms on three different zones were defects were detected: lattice distortions and edge dislocations can be recognized. Thus, from this analysis, we could highlight the presence of mismatch-related defects. Note that the electrical activity of such crystal defects remains to be determined. But given that this low temperature epi-Si on GaAs material contains a large amount of hydrogen (0.1–1%)^29^, a significant fraction of those defects may be passivated.

### A sharp chemical interface

**Figure 4:**
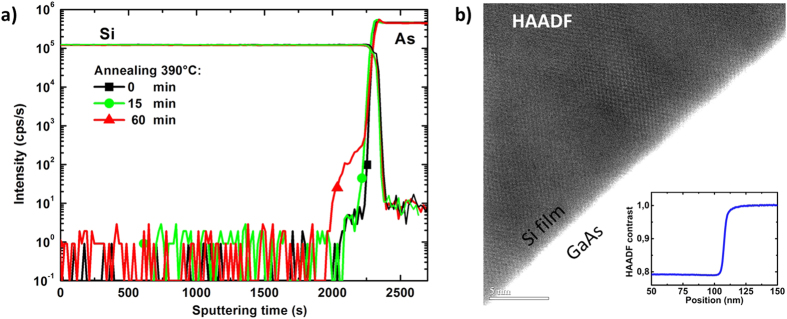
(**a**) SIMS profiles of Si and As across the PECVD epi-Si (1450 nm)/GaAs interface. The profiles are measured for the same sample before (squares) and after annealing at 390 °C in air for 15 min (circles) and 60 min (triangles). (**b**) STEM HAADF image of the interface with the inset showing intensity profile across the epi-Si/GaAs interface.

The epi-Si/GaAs interface has been analyzed by SIMS and HAADF to get detailed information on its chemical composition. The SIMS Si and As profiles are shown in (Fig. 4a), for as deposited and annealed epi-layer. When no annealing is performed (black squares) the transition between Si and GaAs is very sharp, no diffusion is detected. The same sample has been analyzed after 15 min (green circle) and 60 min (red triangle) annealing steps in air at 390 °C. With these latter two annealing conditions, a diffusion profile starts to be visible for As inside silicon. The diffusion of As in silicon may result in n-type doping since As is a donor impurity for silicon. However, in many III-V back end of line annealing processes (metal contacts, etc.) the steps are usually no longer than a few minutes, at temperature in the range of 300 to 400 °C. Consequently, as expected for this low temperature approach, the diffusion across the interface is extremely limited.

Additionally, STEM-HAADF analysis has been performed on the cross section of epi-Si/GaAs samples prepared by Focused Ion Beam (FIB). HAADF images are formed by collecting high-angle scattered electrons with an annular dark-field detector in scanning TEM. Using this imaging method, there is a strong dependence of STEM image intensity on average atomic numbers of the scatterer elements encountered by the incident probe. Thus, a region with lighter elements or simply less atoms will appear darker on a HAADF image; and if the sample thickness is uniform, the HAADF contrast is a function of the material density/chemical composition. A 152 nm epi-Si on GaAs high magnication HAADF picture is shown in (Fig. 4b), together with the intensity profile across the interface shown in inset. In (Fig. 4b) inset, the line scan showing HAADF normalized intensity (with respect to the wafer), corresponds to a 100 nm line perpendicular to the interface in the middle of (Fig. 4b) picture, and averaged laterally on 5 nm. The Si appears darker as a consequence of its smaller atomic number. The crystalline network becomes clearly visible. At this scale, the interface looks again sharp and well defined. Thus this high resolution HAADF picture of the interface confirms the chemically sharp transition as expected from SIMS analysis.

### Relaxed silicon epi-layer

**Figure 5:**
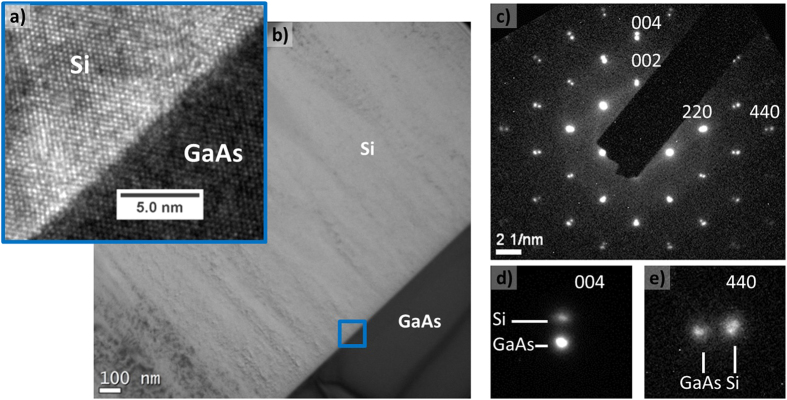
(**a**) HRTEM image along [110] axis of epi-Si/GaAs interface. (**b**) Low magnification cross section picture of a 650 nm thick epi-Si layer on GaAs. (**c**) Diffraction pattern of PECVD epi-Si/GaAs interface: the double points visible for each reflection (e.g. see zooms (**d**,**e**)) are the signature of both Si and GaAs lattices.

To study the strain state of low temperature PECVD heteroepitaxial Si on GaAs, both TEM and XRD analysis were performed. A high resolution TEM analysis of a 650 nm epi-Si on GaAs sample observed in cross section along the [110] axis is detailed in (Fig. 5a), with well defined atomic order visible. The whole layer is visible on the low magnification picture in (Fig. 5b). The electron diffraction pattern acquired at the interface is displayed in (Fig. 5c), and a zoom on 004 and 440 reflections can be seen in (d) and (e). A two points spot pattern can be clearly distinguished, which in fact corresponds to the contribution of Si and GaAs different lattice parameters. This double pattern confirms that the epi-Si growth is not pseudomorphic: from the interface the silicon is growing with a lattice parameter which differs from the one of GaAs, at least partially relaxed. The Si diffraction spots exhibit similar FWHM compared to PECVD epitaxial Si on c-Si wafer, thus suggesting a crystal quality similar to the one obtained with the low temperature PECVD Si homoepitaxial case.

**Figure 6:**
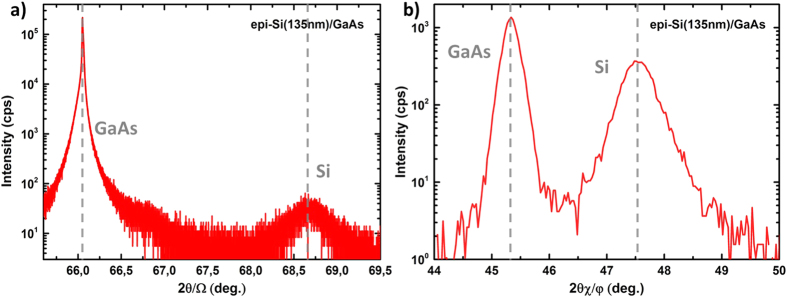
(**a**) High resolution XRD measurement of reflection from the PECVD (004) planes parallel to the surface of the epi-Si film on (001)-oriented GaAs. (**b**) Grazing incidence XRD measurement of reflections from the {220} planes perpendicular to the surface, measured on the same sample.

In addition to the TEM analysis, we coupled high angle 2*θ*/*ω* X-ray diffraction and grazing incidence X-ray diffraction (GIXRD) measurements on a epi-Si(135 nm)/GaAs, grown by low temperature PECVD epitaxy, to get the epi-layer lattice parameter. This set-up allowed us to study the diffraction from the {004} crystallographic planes parallel to the surface (lattice parameter: *a*_⊥_), with 2*θ*/*ω* scan, and the diffraction from the {220} crystallographic planes perpendicular to the surface (lattice parameter: *a*_||_), with 2*θχ*/*ϕ* scan. The Ω and 2*θ* grazing angles were both 0.28°. Additionally, 4 scans were performed along the {220} planes to check the epitaxy relaxation; the absence of twins in epi-Si was also confirmed by large angular scans revealing no peak from {114} planes perpendicular to the sample surface. Figure 6a shows the 2*θ*/*ω* scan with diffraction from the {004} planes, and (Fig. 6b) shows the GIXRD scan with diffraction from the {220} planes. In both cases, the peaks of GaAs substrate and Si epi-layers appear at distinct angular positions; thus confirming the difference in lattice parameters (metamorphic growth). For the 004 reflection, the Si peak intensity is much lower than GaAs: this is essentially due to the small thickness of the epitaxial crystal but also to a lower crystal quality compared to the substrate. In GIXRD, the 220 Si peak appears well-defined with an intensity closer to the one of GaAs, as the X-ray path in Si is much longer for this geometry. Knowing the distances of {004} and {220} GaAs substrate planes, we could deduce from the peaks position the in-plane 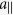 and out-of-plane *a*_⊥_ lattice parameters of epi-Si; we found: 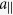 = 5.4049 Å and *a*_⊥_ = 5.4537 Å. The small in plane compressive strain detected is probably more related to epi-Si point-defect content (such as vacancies and hydrogen content forming hydrogen platelets) than to the lattice mismatch with GaAs. A bulk equivalent lattice parameter^30^ of 5.4325 Å for epi-Si on GaAs is found, which corresponds to a difference smaller than 0.1% with standard bulk c-Si. In fact, the mismatch between Si and GaAs is so high that Si relaxes immediately (in few nm) at the interface, and the epi-Si layer is then growing with its own lattice parameter.

## Conclusion

In summary, we have first shown that the native oxide of GaAs substrate can efficiently be removed using SiF_4_ plasma treatment at 175 °C while maintaining the surface RMS roughness below 1 nm. We were able to tune this treatment thanks to *in-situ* surface monitoring by ellipsometry. The subsequent hetero-epitaxial growth of silicon on GaAs has been performed in the same PECVD reactor, keeping a constant temperature of 175 °C, with SiH_4_/H_2_ gas precursors. We found that silane dilution was the key parameter for promoting epitaxial growth: high dilution lead to microcrystalline silicon on GaAs, low dilution resulted in amorphous growth while epitaxial growth was observed for intermediate silane dilutions. The crystal quality of PECVD-Si layers on GaAs grown with the optimum plasma conditions has been thoroughly investigated by TEM and XRD. The cross section TEM analysis has confirmed the absence of oxide a the interface and the excellent crystal order propagating across the interface, thus bringing the proof of low temperature hetero-epitaxial growth of Si on GaAs. We could detect the presence of defects such as misfit dislocations or lattice distortion by using Fourrier filtering of the HRTEM images. From the electron diffraction patterns taken at PECVD-Si/GaAs interface, we could qualitatively see that silicon is relaxed directly at the interface, and this was quantitatively confirmed by a bulk lattice parameter deduced from XRD scans. Moreover, the SIMS and STEM-HAADF investigations have demonstrated the absence of cross diffusion and a chemically sharp Si/GaAs interface. Those results open a new way for combining III-V and silicon which unlocks the main issues encountered in the classic III-V on Si epitaxial growth approach and enable the development of innovative photovoltaic devices.

## Methods

The (001)-oriented GaAs wafers were loaded into the PECVD chamber without any surface pre-treatment, and about 20 min waiting time was necessary for thermalization and outgassing, and to reach a base vacuum around 5.10^−7^ mbar. Using both *in-situ*/*ex-situ* ellipsometry and AFM measurements, the optimum plasma conditions for efficient GaAs native oxide removal were identified: 20 sccm of SiF_4_, 250 mTorr and 0.15 W/cm^2^ for 200 s, with an electrode gap of 22 mm and a substrate temperature of 175 °C. After this native oxide cleaning step, SiH_4_/H_2_ gas precurors were used to deposit silicon layers in the same plasma chamber. By means of ellipsometry and Raman measurements, the optimum plasma parameters for low temperature Si PECVD epitaxy on GaAs were determined: 34 sccm of SiH_4_ and a SiH_4_/(SiH_4_ + H_2_) flow ratio of ~6%, 175 °C substrate temperature, 2.2 Torr and 0.05 W/cm^2^; the corresponding epitaxial growth rate was 1.3 Å/s. The samples were prepared for transmission electron microscopy by FIB method and observed with a JEOL 2200FS TEM (equipped with a spherical aberration corrector for the probe-forming system). High angle 2*θ*/*ω* X-ray diffraction and grazing incidence X-ray diffraction measurements on epi-Si/GaAs samples were performed with a Rigaku smartlab diffractometer.

## Additional Information

**How to cite this article**: Cariou, R. *et al*. Low temperature plasma enhanced CVD epitaxial growth of silicon on GaAs: a new paradigm for III-V/Si integration. *Sci. Rep*. **6**, 25674; doi: 10.1038/srep25674 (2016).

## References

[b1] Tanabe K., Watanabe K. & Arakawa Y. III-v/si hybrid photonic devices by direct fusion bonding. Sci. Rep. 2, 349 (2012). 22470842 10.1038/srep00349 PMC3317235

[b2] Masuko K. . Achievement of More Than 25% Conversion Efficiency With Crystalline Silicon Heterojunction Solar Cell. IEEE J. Photovolt. 4, 1433–1435 (2014).

[b3] Green M. A., Emery K., Hishikawa Y., Warta W. & Dunlop E. D. Solar cell efficiency tables (version 46). Prog. Photovolt. Res. Appl. 23, 805–812 (2015).

[b4] Connolly J. P., Mencaraglia D., Renard C. & Bouchier D. Designing III–v multijunction solar cells on silicon. Prog. Photovolt. Res. Appl. 22, 810–820 (2014).

[b5] Derendorf K. . Fabrication of GaInP/GaAs//si solar cells by surface activated direct wafer bonding. IEEE J. Photovolt. 3, 1423–1428 (2013).

[b6] Umeno M., Kato T., Egawa T., Soga T. & Jimbo T. High efficiency AlGaAs/Si tandem solar cell over 20%. Sol. Energy Mater. Sol. Cells 41–42, 395–403 (1996).

[b7] Lang J., Faucher J., Tomasulo S., Yaung K. & Lee M. Comparison of GaAsP solar cells on GaP and GaP/Si. Appl. Phys. Lett. 103, 092102 (2013).

[b8] Grassman T. . MOCVD-grown GaP/Si subcells for integrated III-V/Si multijunction photovoltaics. IEEE J. Photovolt. 4, 972–980 (2014).

[b9] Kroemer H., Liu T.-Y. & Petroff P. GaAs on si and related systems: Problems and prospects. J. Cryst. Growth 95, 96–102 (1989).

[b10] Kawanami H. Heteroepitaxial technologies of III-V on si. Sol. Energy Mater. Sol. Cells 66, 479–486 (2001).

[b11] Bolkhovityanov Y. B. & Pchelyakov O. P. GaAs epitaxy on si substrates: modern status of research and engineering. Phys. -Uspekhi 51, 437 (2008).

[b12] Fischer R. . Growth and properties of GaAs/AlGaAs on nonpolar substrates using molecular beam epitaxy. J. Appl. Phys. 58, 374–381 (1985).

[b13] Georgakilas A. . Effects of si(100) tilting angle and prelayer conditions on GaAs/Si heterostructures. Appl. Surf. Sci. 102, 67–72 (1996).

[b14] Luxmoore I. J. . III–v quantum light source and cavity-QED on silicon. Sci. Rep. 3, 1239 (2013). 23393621 10.1038/srep01239 PMC3566596

[b15] Yamaguchi M. & Amano C. Efficiency calculations of thin-film GaAs solar cells on si substrates. J. Appl. Phys. 58, 3601–3606 (1985).

[b16] Ringel S. . Single-junction InGaP/GaAs solar cells grown on si substrates with SiGe buffer layers. Prog. Photovolt: Res. Appl. 10, 417–426 (2002).

[b17] Nguyen Thanh T. . Structural and optical analyses of GaP/Si and (GaAsPN/GaPN)/GaP/Si nanolayers for integrated photonics on silicon. J. Appl. Phys. 112, 053521–053521-8 (2012).

[b18] Roesener T., Klinger V., Weuffen C., Lackner D. & Dimroth F. Determination of heteroepitaxial layer relaxation at growth temperature from room temperature x-ray reciprocal space maps. J. Cryst. Growth 368, 21–28 (2013).

[b19] Lee K. H., Jandl A., Tan Y. H., Fitzgerald E. A. & Tan C. S. Growth and characterization of germanium epitaxial film on silicon (001) with germane precursor in metal organic chemical vapour deposition (MOCVD) chamber. AIP Adv. 3, 092123 (2013).

[b20] Dimroth F. . Comparison of direct growth and wafer bonding for the fabrication of GaInP/GaAs dual-junction solar cells on silicon. IEEE J. Photovolt. 4, 620–625 (2014).

[b21] Cariou R. & Labrune M. & Roca i Cabarrocas, P. Thin crystalline silicon solar cells based on epitaxial films grown at 165 °c by RF-PECVD. Sol. Energy Mater. Sol. Cells 95, 2260–2263 (2011).

[b22] Bruneau B. . Ion Energy Threshold in Low-Temperature Silicon Epitaxy for Thin-Film Crystalline Photovoltaics. IEEE J. Photovolt. 4, 1361–1367 (2014).

[b23] Cariou R., Tang J., Ramay N., Ruggeri R. & Roca i Cabarrocas P. Low temperature epitaxial growth of SiGe absorber for thin film heterojunction solar cells. Sol. Energy Mater. Sol. Cells 134, 15–21 (2015).

[b24] Cheng C.-W. . Epitaxial lift-off process for gallium arsenide substrate reuse and flexible electronics. Nat. Commun. 4, 1577 (2013). 23481385 10.1038/ncomms2583

[b25] Lee K., Zimmerman J. D., Xiao X., Sun K. & Forrest S. R. Reuse of GaAs substrates for epitaxial lift-off by employing protection layers. J. Appl. Phys. 111, 033527–033527-6 (2012).

[b26] Hamon G. . Investigation of hybrid tunnel junction architectures for III-V/Si tandem solar cells. EU PVSEC Proc. 1CO.10.4, 75–79 (2015).

[b27] Lachaume R. . Performance analysis of AlxGa1-xAs/epi-Si(Ge) tandem solar cells: A simulation study. Energy Procedia 84, 41–46 (2015).

[b28] Roca i Cabarrocas P., Cariou R. & Labrune M. Low temperature plasma deposition of silicon thin films: From amorphous to crystalline. J. Non-Cryst. Solids 358, 2000–2003 (2012).

[b29] Cariou R. . Ultrathin PECVD epitaxial si solar cells on glass via low-temperature transfer process. Prog. Photovolt: Res. Appl. doi: 10.1002/pip.2762 (2016).

[b30] Labrune M. . Epitaxial growth of silicon and germanium on (100)-oriented crystalline substrates by RF-PECVD at 175 °C. EPJ Photovolt. 3, 30303 (2012).

